# Supplementary material for: Increased performance of DNA metabarcoding of macroinvertebrates by taxonomic sorting
Source: PLoS One. 2019 Dec 16;14(12):e0226527. doi: 10.1371/journal.pone.0226527 (PMC6913968; doi:10.1371/journal.pone.0226527)
Supplement: Attachment — Submitted filename: Response to Reviewers.docx [file pone.0226527.s006.docx]

**Response to reviewers**

We would like to thank the editor and reviewers for considering our submission, and for their constructive feedback on the manuscript. We have incorporated some changed based on their suggestions in the revised manuscript.

One of the main issues was the lack of clarity regarding the rarefaction of data we performed to address the uneven sequencing depths between pooled and sorted samples. We have added a paragraph to the methods section detailing our approach.

While we can understand the concerns about the primer choice by reviewer #2, we wanted to perform amplifications with a single set of primers, mostly since we are trying to work towards a standard way of processing monitoring samples that should not be more complicated and time consuming than the current morphological assessments. By our knowledge, the primers selected perform well on a wide variety of macroinvertebrates, both in theory and in practice. We have assessed several primer sets prior to this project, and these primers showed most potential across the different taxonomic groups. We have included a statement about the primers in the discussion.

Based on suggestion by reviewer #1, we have combined the data from figure S4 into figure 3. This was certainly a useful suggestion, as both figures displayed similar information. Figure S4 has been changed to only detail the comparison of EQR scores with and without water mites, as that group was not included in the DNA analysis. We have also changed figure 2, to also provide the lower error bars, and we have removed the values of the fractions listed in the figure.

A few minor other edits were made to the manuscript and the figures and their legends to address the other concerns of the reviewers, and have provided a response to the comments by the reviewers below.

**Review Comments to the Author**

**Reviewer #1:** The authors present a methodological analysis of DNA metabarcoding, using a set of 25 freshwater arthropod community samples, with a particular focus on taxonomic composition of samples. They present a comprehensive pipeline by which they identify their OTUs to species level, using this data to compile molecular species lists and community indices. They have particular power in this analysis as these samples have been previously sorted and morphologically identified, against which data the authors validate their species lists and diversity/composition indices. Their main methodology is the separate DNA metabarcoding of separate taxonomic groups within each sample, compared against DNA metabarcoding of all of these groups combined within each sample. Their metabarcoding results suggest that in general, morphological and molecular species lists have considerable overlap, but metabarcoding misses a substantial proportion of species. Their key conclusion is that this observation is exacerbated in pooled samples containing all taxa, even when rarefying to ensure standardisation of effective sequencing coverage, and thus that metabarcoding taxa separately is important for full community recovery.

This research is largely well presented, although I feel the depth of analysis is somewhat shallow in places. In particular, the authors do not analyse or discuss the possible effect of sequencing depth on their findings, both in terms of the extent to which they can compare between samples and the extent to which their findings are able to be generalised. While they present the total numbers of reads recovered from sequencing, they only passingly report some of the post-filtering and bioinformatics per-sample reads and ranges. Rarefaction is only passingly discussed (in the results section), and the reader's understanding of sample treatment is confounded by unclear description of the way that PCR replicates were combined. While it is clear, and largely justified, that the authors rarefied the read counts of the pooled samples to be equal to 6 times those of the separate-taxon samples, it is not stated what these rarefaction targets were, nor unequivocally stated whether all separate-taxon samples were rarefied to the same target. I am sure the authors agree that this is a crucial step if comparing lists of recovered species or summary indices. I suggest that the authors clarify how exactly technical replicates are treated as well, as it is unclear whether data from these were combined pre- or post- rarefaction. The authors' description of the 'additive' combination of PCR replicates implies that only species lists were combined; if so, this is somewhat concerning given they report several sequencing failures - I don't feel that species lists from samples with two successful replicates would necessarily be comparable to samples with only one. A more valid approach would see the data from multiple replicates being combined before each independent sample is rarefied to the same rarefaction target (or discarded for having lower than the target number of reads). Finally on this, the authors report that some of the source 25 samples did not have all six taxa: should the pooled samples from these not be rarefied to the appropriate lower multiple to make this more comparable?

Our response: W thank the reviewer for pointing out the lack of clarity in our rarefaction approach. We have included a paragraph in the methods section describing the rarefaction targets for the samples that were used for the comparison with rarefied data. The reviewer suggests we combine replicates before rarefaction, which is what we have done in the original manuscript. As stated before, we have elaborated more on the rarefaction procedure in the methods section of the paper. We choose to rarefy all combined and sorted samples to the same depths. We rarefied all pools to the lowest read depth available, and rarefied all separately sequenced samples to one sixth of that. We did not opt to rarefy to a lower multiple for samples with missing taxa. While this may have provided more exact comparison, the increased richness from separately sequenced taxon groups presented in the paper is in our cases still higher than the pooled sample, even with lower read depth! The increase in the richness would probably have been a little higher than the reported 2.22 times, but there is already no significant difference between the increase in the rarefied and non-rarefied datasets.

More widely, beyond simply reporting per-sample read numbers and rarefaction targets, the authors could improve the extent to which their results can be generalised by examining the effect of different read numbers on their findings - assuming they have sufficient coverage in the first place. This could be achieved by utilising a range of rarefaction targets to simulate reduced sequencing coverage, and examining how the values presented in figure 2 respond. This could all be simulated based on existing data. The authors may be interested in a recent paper (doi: 10.1111/1755-0998.13008) that suggests that protocol variation can have substantial effects on community recovery if coverage does not reach an asymptote (although not specifically regarding taxon composition)

Our response: While this is an interesting suggestion made by the reviewer, in the current situation the rarefaction targets were already low (15,000 for pools and 2,500 for separate taxon groups). Going any lower will not provide meaningful results, in the scope of the manuscript.

Otherwise, I have no other major comments on this nice paper, but there are various presentational and detail aspects which hinder the reader's easy understanding of the subject matter and the authors' comprehensive discussion of their findings:

Abstract: The authors define WFD (line 20) but not EQR (line 30). "Such as" on line 22 seems misplaced. Otherwise clear and succinct.

Our response: We have defined EQR in the abstract, and have rewritten the sentence on line 22 to make it more clear.

Introduction: The introduction is short, but this is fine, it provides a clear background and well stated aims without waffle. I would suggest that line 46: "Until this day" be changed to "To date" as a more standard English phrase.

Our response: We have changed “Until this day” into “To date”.

Methods:

>Sample selection and processing:

Line 77: Citation needed for "identified according to national WFD monitoring standards" - what are these methods/standards?

Our response: We have included a citation to the national guidelines (albeit a Dutch handbook).

Line 79: Some explanation for the EQR score range and quality classes would improve reader understanding here - at the very least a citation for the reader to find more, but an in-text explanation preferred.

Our response: A brief in-text explanation of the Dutch EQR scoring has been added to the methods section, with a citation to a paper with more explanation on the calculation and classification of the scores.

It is unclear to the non-Dutch reader what exactly Aquon is - in line 76 I assumed it was a specific location, and was confused later that a location was performing identification. It took context clues from later (and a google search) to understand that it was a freshwater ecological surveying company. I suggest reorganising this paragraph to make this clearer: for example, "Freshwater arthropod samples were collected using [method] in [location] by [ecological survey company] Aquon on [dates] according to [standardised WFD monitoring guidelines]. Aquon taxonomists sorted and identified these samples into [groups]. For this study, we selected 25 samples based on ratio ... etc"

Our response: Thanks for pointing out that it was unclear what Aquon was. We have rewritten the paragraph along the lines of the reviewers suggestion, emphasizing that Aquon is an ecological survey company.

It's not crucial, but some useful context could be supplied if the authors report the total number of samples from which the 25 were selected, and more importantly the overall EQR and quality class ranges from which their sample EQR and quality class ranges were decided. It's currently unclear to what extent their samples provide a wide sample.

Our response: We have included some details on the total number of samples and the ranges of EQR and Shannon-index to show that the selection of 25 reflects the overall range of the samples available.

On line 90, the authors report 158 tubes. 25 samples of 6 sample groups used gives a total of 150 - where do the extra 8 come from?

Our response: We have removed this sentence as it was confusing without further elaboration and did not add much to the manuscript. There were initially more tubes than 150, even though some taxon groups were absent from a few samples, as some samples contained more specimens than what fit into a single tube. The multiple tubes of specimens belonging to the same taxon group and the same sample, were combined in the analysis however.

>DNA extraction and amplification:

The authors do not report the number of species, or more crucially the numbers of individuals or the biomass ranges of species within tubes, nor the extent to which this varies between samples of the same taxon or different taxa. Considerable variation in these values may introduce biases to molecular recovery rates and diminish the validity of their findings. The authors mention the effect of biomass on OTU recovery in passing in their introduction, and again in their discussion, but do not report any attempts to mitigate these effects.

Our response: Supplemental File S1 has the full taxa lists of both morphological and molecular analysis, together with specimen counts (or read counts for DNA). We have included a reference to the supplemental file in this part of the text to stress that. We did not assess biomass in detail, but included mention of it in the introduction as it is often reported as a confounding factor in metabarcoding studies. In the discussion we have removed the mention of biomass, as it did not fit in with the rest of the reasoning in that particular part of the text.

>Bioinformatics

This section could be improved by more detail to improve reproducability of the methods. For each step of the bioinformatics process, the authors should fully report the parameters used, or that default parameters were used. Furthermore, there should be some justification for software and parameter choices made. No denoising was performed, and reads shorter than the target length were permitted but not reads longer. Length variants were permitted that allowed frameshift mutant sequences (length variants not a multiple of 3 bases different from the target length) - these are likely to be sequencing errors rather than true sequences. It is not clear at what point sequences were converted from fasta to fastq? It is also unclear whether OTU clustering was performed on a by-sample basis or over the entire dataset.

Our response: Agreed about the parameters. We have added the setting for the merging and primer trimming (even though these might be more or less default). The other settings were already included in the manuscript. We allowed for three bases longer and 3 bases shorter than target length (313 bp). This was to allow for some variation in length that occurs naturally (and has been observed during DNA barcoding of some specimens) and potential primer slippage issues as reported by the authors of the primers used in this study (citation included to manuscript). Clustering was performed over the entire dataset.

Line 161: "Anticipating on" should just be "Anticipating"

Our response: We removed “on”

>Comparison morphology versus DNA

The title of this section is not good English. It should be something along the lines of "Comparison of morphology vs molecular identification"

Our response: We have changed the title.

Line 181: what is meant by "accumulated" in the context of shared taxonomic identity. Does this just mean merged? It would be illuminating if the authors provided some analysis or commentary on what the recovery of multiple OTUs per species says about the OTU clustering methods, particularly whether this varied between taxa

Our response: We changed “accumulated” into “aggregated”. We did indeed add up all the MOTUs that had identical taxon identification. Multiple MOTUs per species is only in part due to the clustering method employed. Differences in intraspecific variation between different taxa is a more important factor, which inherently leads to either overestimations of diversity for taxa higher intraspecific diversity (or underestimations by lumping of closely-related taxa with low interspecific variation if cluster settings are not strict enough).

Line 182: Again, what is meant by "accumulated" in this context?

Our response: The use of this word for two different processes was indeed confusing. We added together the taxa lists of the sorted samples. We have rephrased this sentence for clarity.

There could be a little more discussion of ecological quality ratios: what are these actually measuring? Was morphological data also converted to presence-absence? Studies into methodological considerations in metabarcoding are relevant to ecologists from a wide range of backgrounds, not just those working in freshwater or marine monitoring. Some of this is discussed in more detail in the discussion, but until then a reader unfamiliar with EQRs is left in the dark.

Our response: We have included a short explanation of EQR and some citations to relevant literature. Morphological data was also converted to p/a, we have updated the text to reflect that.

Results

As supplied, having figure captions in the text but the figures at the end of the document is a bit of a pain to read. It would be easier to have the figures in the text, or at least the captions with the figures at the end.

Our response: This was done according to PLOS ONE guidelines.

The results are by large comprehensively and clearly reported. There is a substantial amount of detail here that is perhaps unnecessary, and the reader can easily lose track of the main finding, the comparison between the combined taxon-specific samples and the pooled samples in terms of recovered community composition. In particular the reportage of taxonomic composition is a bit dry and repetitive, and figure one is not particularly surprising or informative. Perhaps the authors could find a more engaging way to present taxonomic composition and move figure one to the supplement - entirely their choice though.

Figure two as supplied is portrait and difficult to read. Furthermore, it appears to be missing the lower standard error bars? The response axis should read richness rather than diversity, to correspond with the language in the text and be unequivocal.

Our response: The automated system has rotated the figure for some reason, it is not submitted nor supposed to be in this orientation. Error bars were the same in both directions and we omitted the lower error bar for esthetic reasons. We have, however, put them back in to avoid confusion.

Line 267 is missing a closing parathesis

Our response: Added closing parenthesis.

The rate of uncertainty in taxonomic identification at both morphological and molecular level is unsurprising, and the authors should be applauded for undertaking considerable work in designing a pipeline to achieve the best possible molecular identifications. However, this does beg the question: to what extent might the findings be different if the authors focused only on species/OTUs that can be definitively identified to species level by both morphological and molecular methods. I.e. rather than What happens if drop out comparisons of presence/absence of any uncertain taxon, and focus solely on clearly identified species at both DNA and morphological level? I.e. the dropping of any entry in category 2-4 from the lines 191-200. At very least, it would be useful if the authors could report the number of entries falling into each category to reassure the reader that uncertainty levels were low

Our response: The majority of the matches were species level matches in both lists (72,6%), with only 5.1% flagged as potential misidentifications. The remainder were matches that were not on the same taxonomic level due to missing references (mostly annelids) or deeper identifications with barcode data (mostly dipterans). We have added a section to the results detailing these findings.

I would suggest bringing Supplemental Figure 4 into the main text, this quite strikingly shows the contribution of the annelids to the variation in EQR scores between Fig 3B and 3C-H. The authors may wish to consider simplifying and perhaps even merging Figures 3 and 4 by removal of the coloured blocks and simplification of axes etc.

Our response: We have combined Figure 3 and Figure S4 into one figure and simplified the layout for clarity.

Discussion

The discussion is detailed, thoughtful and well referenced, with the conclusions well supported by the results. It could perhaps be reduced in length somewhat.

In line 364, the authors report that size-based sorting affects recovery rates, yet they do not justify why they did not do this.

Our response: We intended to refer to another paper that performed pre-processing (size sorting) before DNA extraction, and we wanted compare our findings to theirs. Our focus was on taxonomic sorting. Both taxonomic and size sorting may improve taxon recovery even more, but would require more pre-processing time.

The authors do not compare their findings to recent work also looking at the effects of taxonomic composition on OTU recovery in metabarcoding, which did not use morphological data as a comparison but instead used pools of increasing taxonomic complexity, finding somewhat similar results (doi: 10.1002/ece3.4839).

Our response: Thanks for pointing out this interesting paper. We’ve updated the text in several places to refer to this paper where it concerns discussions about primer bias. We’ve also added a few sentences to the discussion comparing the findings of this paper to ours.

I congratulate the authors on an interesting paper, and am sure it will be an important contribution to metabarcoding and molecular ecology with the above comments addressed.

**Reviewer #2:** The authors present an interesting paper evaluating the performance of metabarcoding versus morphological assessment of aquatic invertebrate samples. Although the general premise of the paper is interesting, I have a few concerns on the methodological and technical aspects of the paper. My first concern is the use of only a single primer set (BF1/BR2) to assess a community not only containing insects, but crustaceans, annelids, and molluscs. This primer is not well suited for organisms from different phylums and I would argue that is a poor primer choice for non-insects. The poor performance of this primer pair on the pooled sample is unsurprising given the diversity of organism (or templates) present in the samples.

Our response: To our knowledge, the primers used in this study are one of the best for amplification of freshwater macroinvertebrares, as they were specifically designed for this purpose. The original paper presenting these not only looked at insects, but other taxonomic groups as well. While it’s true that their mock community was mainly insects, the in silico assessment indicated a good performance for many other groups, including all groups included in our study, especially compared to some other oft-used primers. Since the reviewer doesn’t make any suggestions for alternate primers, it is impossible for us to assess the effects of using a different primer set. The fact that some groups are not recovered well in the pooled samples is more due to preferential amplification of other groups.

My other major concern is that the authors try to account for uneven read depth by taking one six of the read depths to create pseudoreplicates of pooled samples from sorted samples (at least that is my understanding from the text). The simplest solution and most comparable solution would be to subsamples reads to have an equivalent read depth for all samples. This would allow a more meaningful comparison of recovery amongst all the samples.

Our response: Thanks for pointing out that the rarefaction was confusing. We’ve updated the methods section to provide information on the rarefaction. We hope it is now clear what we intended to do. We rarefied all pooled samples to the lowest read depth available (15,000) and all sorted samples to 1/6^th^ of that so that they would have the same read depth as the pool when combined.

My third major concern is that text can be a bit confusing on the discrepancies between morphological and molecular identifications. I think it would be best to clearly state the errors in either data set (preferably morphology first) and then elaborate on the where mistakes are made in the other approach. I.e. I want to see some sort of assess of the quality of morphological ids and the reference library. This will make it easier to then sort out differences in recovery between the two approaches.

Our response: While we agree, assessing the errors in the reference database without having all identifications checked manually by taxonomic specialists is almost impossible. It is a given that any publicly available database will have errors, which in itself is topic of much debate surrounding the use of these databases. The idea behind our study was to take the morphological data “as is” and compare it to the DNA metabarcoding results. We refrain from calling either of the two (in case of mismatches) “wrong”. In the end, most of the entries that were “matched” in both datasets were direct species matches (72,6%), and only in 5% of the cases did we flag something as “potential misidentification” when both lists had different species from the same genus. We could not re-evaluate the specimens in these cases, as they were all homogenized for DNA extraction. We adapted the manuscript to include these percentages, as an indication of the level of certainty for the matches between morphology and metabarcoding data.

Otherwise, I did appreciate the authors use of the top 100 hits to assign taxonomy to MOTUs. This is a great approach that will hopefully become more common in the field and is a strength of the paper. I have a few other comments below:

Line 56: could probably add a few more references for mock communities and maybe argue that there is a greater need to use actual samples because they tend to be a bit naïve. I.e. the diversity present in your samples is a testament to the limitations in diversity often present in mock samples.

Our response: We included some more references to mock community studies here.

Line 100 – 102: This sentence is quite awkward!

Our response: This sentenced has been rephrased for clarity.

Lines 92 – 102: why was no estimate of DNA concentration done prior to pooling? Depending on extraction efficiency this could have introduced another massive bias. It would have also allowed you to assess the quality of samples following extraction because what if one community was more degraded then another?

Our response: This was intended. By pooling based on volume instead of DNA concentration, we simulated a situation in which all taxa were extracted simultaneously. Some taxon groups represented larger proportions of biomass, and we wanted this to be reflected in the pools. We assumed all DNA was equally degraded, freshly collected samples were all treated and stored in the same conditions.

Lines 116 -130: This cannot be changed after the fact but why so many PCR cycles? Also the use of AIC was great in addition to extraction blanks!

Our response: We agree that fewer PCR cycles might have been better, in this case, however, we followed a protocol we used for prior publications (doi: 10.7717/peerj.7335).

Line 142-144: Although I am good with using an additive approach, it could be argued that some of these are stochastic in nature and this approach can over count OTUs. I think this approach can be justified and maybe a sentence or two would be useful.

Our response: While it increases the number of MOTUs (and thereby overestimates richness), our intent was to recover as many taxa as possible. In the end, we looked mostly at taxa, not at MOTUs. We’ve added a sentence to stress this.

Line 146-159: Why not query and remove contaminants as the first step? And why not screen and remove them from your reference library first?

Our response: Very true, common contaminants like human or Wolbacchia could have been filtered prior, but there could be many other forms of contamination that are not so easy to account for, especially if you are uncertain of what to expect. This is exactly why we screened against two databases, to detect any contaminant issues that were not so glaringly obvious as human sequences. More importantly, there were also cases in which there wasn’t necessarily a contaminated sequence in the reference, but weaker matches were found to macroinvertebrate reference database sequences when the actual query sequence was a contaminant (not a macroinvertebrate). In these cases, checking with references that included non-macroinvertebrates made it possible to identify these sequences as such.

Figure 1: What exactly is the difference between DNA taxon richness versus MOTU richness. I think there is a mistake in the caption that should be reviewed. I am assuming one is supposed to be morphological richness? I would just review terminology through the material and methods as well as the results.

Our response: We plotted both the number of MOTUs and the number of taxa found in metabarcoding against the number of taxa found in morphological analysis. The discrepancy between number of MOTUs and number of taxa is due to several taxa being represented by multiple MOTUs, which is explained in the discussion.

Figure 2: have “observed diversity observed” maybe change the second observed to present?

Our response: Changed to “present”.

Line 298: So the results are mutually exclusive, which on was correct or are they both wrong?

Our response: We refrained from making any statements as to which of the two identification was wrong. It is quite possible they are both correct (in samples with species that were not recognized during morphological assessment, as well as species which failed to amplify).

Line 351-353: what were the quality of hits?

Our response: We are unsure what the reviewer means by quality of the hits. The LCA tool used matches that were around 90% with species from this genus. None of the species in the morphological list were present in the reference, but some non-Dutch congeners were, which were picked up by the LCA. We only found this genus in DNA data for samples in which species were present in the morphology data, so even though the matches are only around 90%, we can be fairly certain these are correct.

Lines 435-439: I would argue that this is due to poor primer choice for this group.

Our response: While it may not have been the most optimal primers for molluscs, we would argue that molluscs are the group that is most affected by differences in biomass between the different species. General primers are by definition sub-optimal and there are always trade-offs, but we still amplify many different molluscs. In any case, the idea behind the study was to perform all amplifications with the same primers, and to our findings in literature and some preliminary testing, the selected primers performed best across the different taxa.

Conclusions: I agree that things are not always clear cut but can you make a more concrete statement. Does metabarcoding provide reliable data for EQR scores or do both approaches have fundamental flaws

Our response: We have added a statement to the conclusion. While we cannot say that either of two methods is “wrong” in case of mismatches, at least the use of DNA will allow for more consistent taxon lists, as there is no bias introduced by different assessors with varying levels of expertise.
