# Supplementary material for: Increased performance of DNA metabarcoding of macroinvertebrates by taxonomic sorting
Source: PLoS One. 2019 Dec 16;14(12):e0226527. doi: 10.1371/journal.pone.0226527 (PMC6913968; doi:10.1371/journal.pone.0226527)
Supplement: Attachment — Submitted filename: Response to Reviewers V2.docx [file pone.0226527.s007.docx]

**Response to reviewers**

We would like to thank the editor and reviewers for considering our re-submission. We have adjusted the manuscript based on the remaining points raised by the reviewer.

We ran some of the analysis again based on an inconsistency noticed by the reviewer in the length used to trim the sequence data. To be certain we went back to the data and noticed it was not a typo in the manuscript, but we did indeed use incorrect values for trimming. After comparing the new results to the earlier ones, we found only small differences in the MOTU clustering, leading to a few additional MOTUs and changes in the number of reads for a selection of MOTUs. However, we found no additional taxa, other than some MOTUs identified at genera level that were already found at species level, and rare cases vice versa. Since we used a taxonomic aggregation and removed redundant taxa (as described in the manuscript) prior to comparison with morphology, we observed no changes in the analyses beyond the creation of the MOTU table. Please note that we have adjusted the numbers in the manuscript to reflect the new MOTU table with the corrected sequence filtering, and also updated Figure 1 to show the new MOTU richness, as well as supplementary File S1.

We have provided a response to the other comments by the reviewer below.

**Review Comments to the Author**

**Reviewer #1:** Thank you to the authors for providing a detailed response to my comments and supplying a revision of their manuscript. I am satisfied that the vast majority of my concerns have been addressed. I have only relatively minor comments on this revision that is otherwise suitable for publication. Note that my line numbers refer to the tracked changes version of the manuscript.

Firstly, there is inconsistency in the reporting of the fragment length. On line 127 you state the length of the fragment amplified is 316bp, in agreement with Elbrecht and Leese and supplemental figure S1. However, in the response to reviewers you state the length is 313 and that you allowed a +/- 3-base variation in length around this, giving the 310-316bp stated on line 160. Which is it? It seems as if you may have allowed sequences up to 6bp shorter than the target, but no longer. In the slippage paper cited, the forward primer was observed to reduce the length by 1-2 bases, and they did not study the reverse primer. While allowing for primer slippage by using a loose length threshold may retain true biological sequences, it may simultaneously allow PCR or sequencing errors through to create erroneous MOTUs that may inflate your MOTU richness. In my opinion this concern should at least be noted and discussed in the paper, even if the authors choose to retain this less conservative length variation threshold.

Our response: We checked the raw data and we indeed made a mistake in the analysis rather than in the manuscript. We have ran the analysis again as discussed above.

Otherwise, I suggest the authors review the paper for language once again, I noticed relatively frequent typos or slight English errors. For example on line 30 "ration" should be "ratio" and line 60 "organisms groups" is not correct English. Similar minor errors are found throughout the paper. This doesn't detract from the understanding of the paper, simply an aesthetic concern.

Our response: Several mistakes throughout the paper were corrected and sentences were rewritten for clarity.

On line 116, the authors should report the exact speed or frequency of the homogeniser used, rather than stating the maximum was used, as different devices may have different maxima. According to this https://www.ika.com/en/Products-Lab-Eq/Dispersers-Homogenizer-csp-177/ULTRA-TURRAX-Tube-Drive-Technical-Data-cptd-3646000/ it's 6000rpm

Our response: We have added the speed used (indeed 6000 rpm) to the methods section.

I should note that I broadly agree with reviewer 2's comments on primer choice, something I clearly didn't think about enough on my first review. I believe the current methodology provides sufficiently supported and interesting results for publication, however there is certainly some scope for discussion of primer choice. After all, the authors advocate for separate amplification of taxon pools, negating the need for a single general multi-taxon-appropriate primer-pair (or even locus). In the response to reviewers, the authors provide good reasoning for why the primers are not to blame for poor amplification of molluscs, however there seems no reason why such a discussion cannot be also included in the text of the paper itself, to acknowledge that primer choice may have been an source of error in the study, discuss whether this is the case and propose future methodological directions to ameliorate this issue.

Our response: We have added several parts of the discussion in the rebuttal letter on this topic to the main manuscript.

On the other hand, I agree with the authors that their pooling on volume instead of concentration was the appropriate route to take for accurate reflection of a multi-taxon extraction.

Otherwise, the authors have improved the clarity of the text, especially on the issue of rarefaction, and overall the paper reads well, the work is well-justified and results, discussion and conclusion well-supported.
